# Unusual manifestations of chest wall tuberculosis: diagnostic challenges in two cases from a low-incidence tuberculosis country

**DOI:** 10.1093/jscr/rjag890

**Published:** 2026-10-04

**Authors:** Mahmoud Yehya, Bhagwan Mantri, Abubaker S Ahmed, M S Abdelfattah, Mhmad Othman J Albaghdadi

**Affiliations:** Thoracic Surgery, SAQR Hospital, Al Juwais, Al Kadi Street, Ras Al‑Khaimah, United Arab Emirates; Department of Pulmonology and Sleep Medicine, Ibrahim Bin Hamad Obaidullah Hospital, Ras Al‑Khaimah, United Arab Emirates; Department of General Surgery, SAQR Hospital, Al Juwais, Al Kadi Street, Ras Al‑Khaimah, United Arab Emirates; Infection Control, SAQR Hospital, Al Juwais, Al Kadi Street, Ras Al‑Khaimah, United Arab Emirates; Kasr Alainy School of Medicine, Cairo University, Al Saraya St Old Cairo, Cairo Governorate 4240310, Egypt

**Keywords:** chest wall tuberculosis, extrapulmonary tuberculosis, tuberculous lymphadenitis, cold abscess, chest wall mass, diagnostic challenge, anti-tuberculous therapy, case report

## Abstract

Chest wall tuberculosis (TB) is a rare manifestation of extrapulmonary TB and may present a significant diagnostic challenge, particularly in low-incidence TB countries such as the United Arab Emirates. We report two unusual cases of chest wall TB with distinct clinical presentations. The first case presented as a slowly enlarging chest wall mass that clinically and radiologically mimicked a soft-tissue neoplasm and was ultimately diagnosed as giant tuberculous lymphadenitis. The second case presented as a large posterolateral chest wall abscess without evidence of active pulmonary TB. Both patients were managed surgically for diagnostic and therapeutic purposes, and histopathological examination established the diagnosis of chest wall TB. Both patients received a 6-month course of anti-tuberculous therapy with complete recovery. These cases highlight the diverse clinical spectrum of chest wall TB and emphasize that the disease should be considered in the differential diagnosis of chest wall masses and abscesses, even in low-incidence TB settings. Histopathological confirmation remains essential for establishing the diagnosis and guiding appropriate management.

## Introduction

Tuberculosis (TB) remains one of the leading infectious diseases worldwide despite significant advances in diagnosis and treatment [1]. The United Arab Emirates is a low-incidence TB country with an estimated incidence of ~1 case per 100 000 population [2], hence, awareness among clinicians is important to aid recognition, treatment, and avoidable mortality. Although pulmonary involvement is the most common form, extrapulmonary TB accounts for a substantial proportion of cases. Musculoskeletal TB represents a relatively uncommon manifestation, accounting for ~2.6% of all TB cases and 15%–20% of extrapulmonary disease [3].

Chest wall TB is a rare localization of extrapulmonary TB and accounts for 1%–5% of all musculoskeletal TB. The disease may arise through hematogenous dissemination, lymphatic spread from adjacent infected lymph nodes, or direct extension from pulmonary or pleural lesions [1, 3–5]. Clinical manifestations are often solitary, steadily enlarging chest wall mass with or without pain [3]. A painless cold abscess or sinus tract formation may be the only presenting clinical feature for a prolonged period [5].

The diagnosis of chest wall TB remains challenging and requires a high index of suspicion, particularly in the absence of associated pulmonary disease. Because radiological findings are often nonspecific, definitive diagnosis relies on histopathological and microbiological confirmation, typically demonstrating granulomatous inflammation with caseous necrosis and evidence of *Mycobacterium tuberculosis* [3, 5].

Medical therapy alone often fails to treat chest wall TB, and surgical intervention is required in most cases [3].

Herein, we describe two unusual presentations of chest wall TB encountered in our institution. The first case presented as a solitary chest wall mass closely resembling a soft tissue neoplasm and was ultimately diagnosed as giant tuberculous lymphadenitis. The second case presented as a massive, isolated chest wall abscess without radiological evidence of active pulmonary TB. These cases highlight the diagnostic difficulties associated with chest wall TB and emphasize the importance of maintaining a high index of suspicion, even in the absence of pulmonary disease.

## Case presentations

Case 1: A 24-year-old male patient presented with a painless, progressively enlarging lump over the right upper anterior chest wall for 12 months. The patient denied constitutional symptoms including fever, chronic cough, or weight loss. There was no previous history of TB or known TB contact. Physical examination revealed a painless, freely mobile right subclavicular mass measuring ~7 × 7 × 10 cm, with a homogeneous rubbery consistency, stretched overlying skin, and central ulceration associated with serosanguineous discharge (Fig. 1) The remainder of the chest examination was unremarkable, and no peripheral lymphadenopathy was detected.

**Figure 1:**
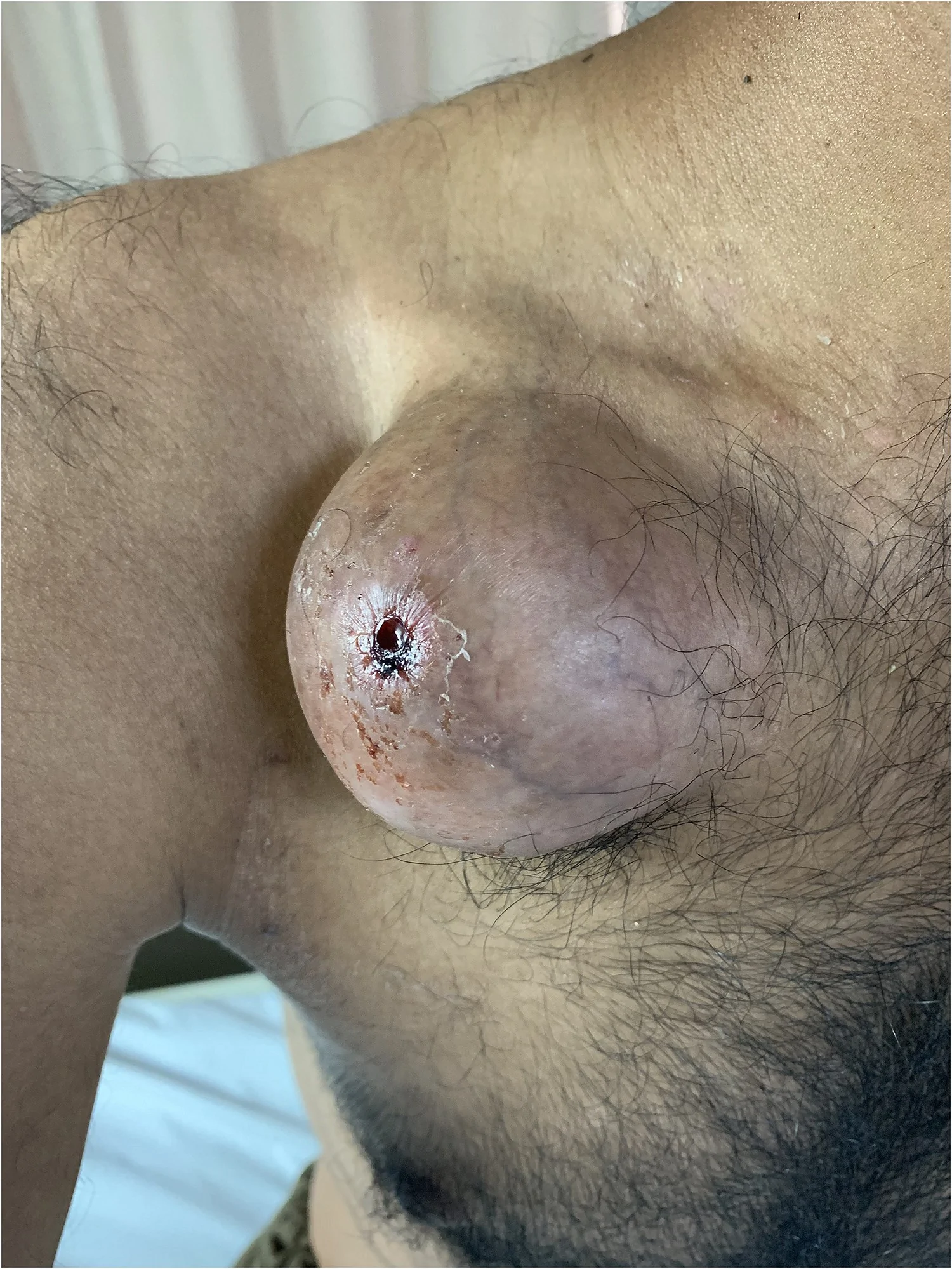
Clinical photograph demonstrating a large, painless, freely mobile mass in the right subclavicular region of the anterior chest wall, measuring ~7 × 7 × 10 cm. The lesion is well circumscribed with stretched overlying skin and central ulceration associated with serosanguineous discharge.

Routine laboratory investigations were unremarkable, apart from an elevated C-reactive protein level (CRP) of 36.7 mg/l (reference range: 0–9 mg/l).

Chest radiography revealed a well-defined large, rounded opacity in the right upper lateral chest wall without associated pulmonary or pleural abnormalities (Fig. 2).

**Figure 2:**
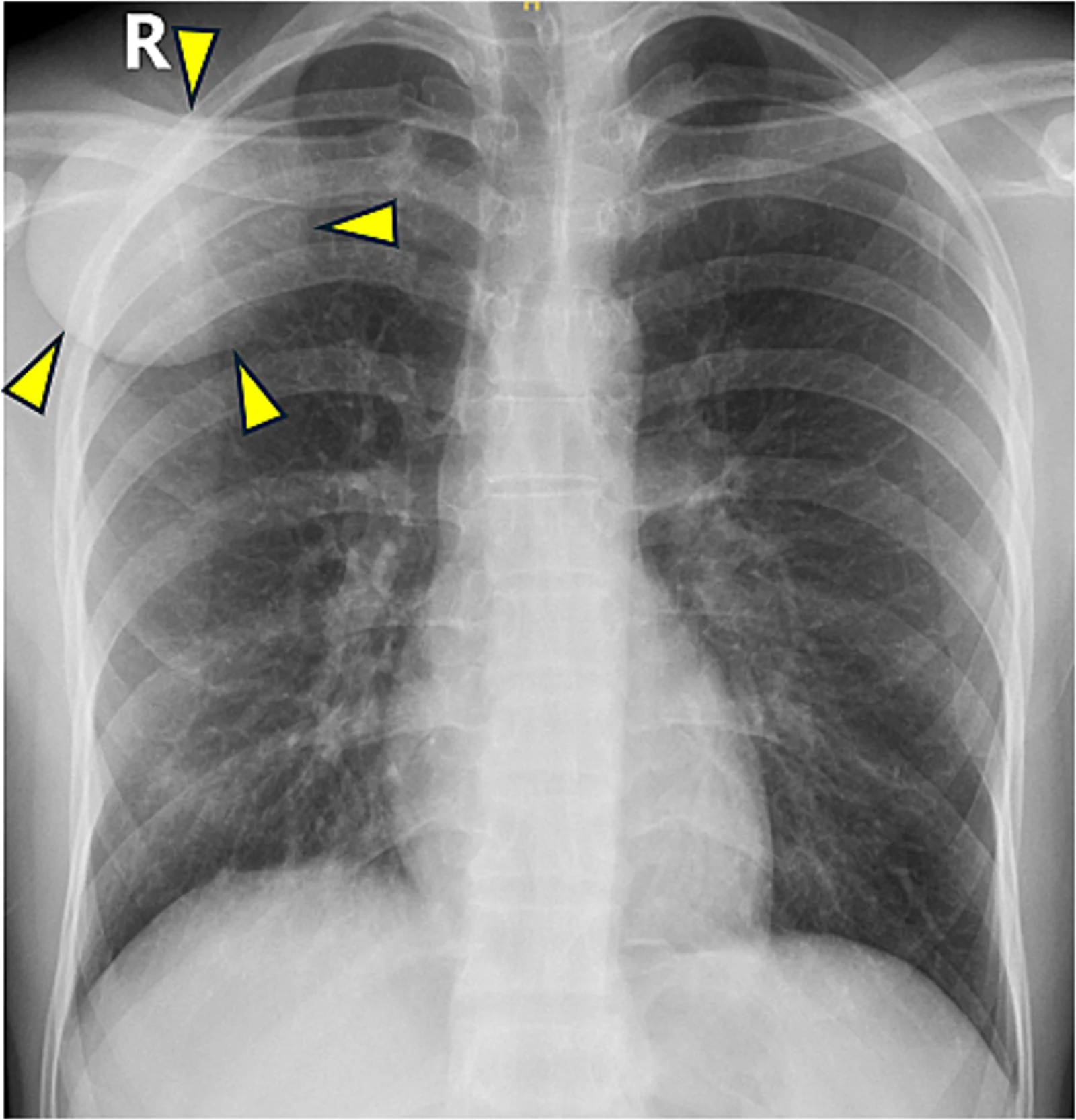
Posteroanterior chest X-ray demonstrating a large, well-defined rounded soft-tissue opacity in the right upper lateral chest wall (arrowheads). No radiographic evidence of active pulmonary or pleural disease is identified.

Computed tomography (CT) of the chest revealed a well-defined encapsulated subcutaneous mass in the right upper anterior chest wall, measuring 75 × 52 × 57 mm, with internal calcifications. The lesion was separate from adjacent thoracic structures, and no pulmonary lesions or mediastinal lymphadenopathy were identified (Fig. 3).

**Figure 3:**
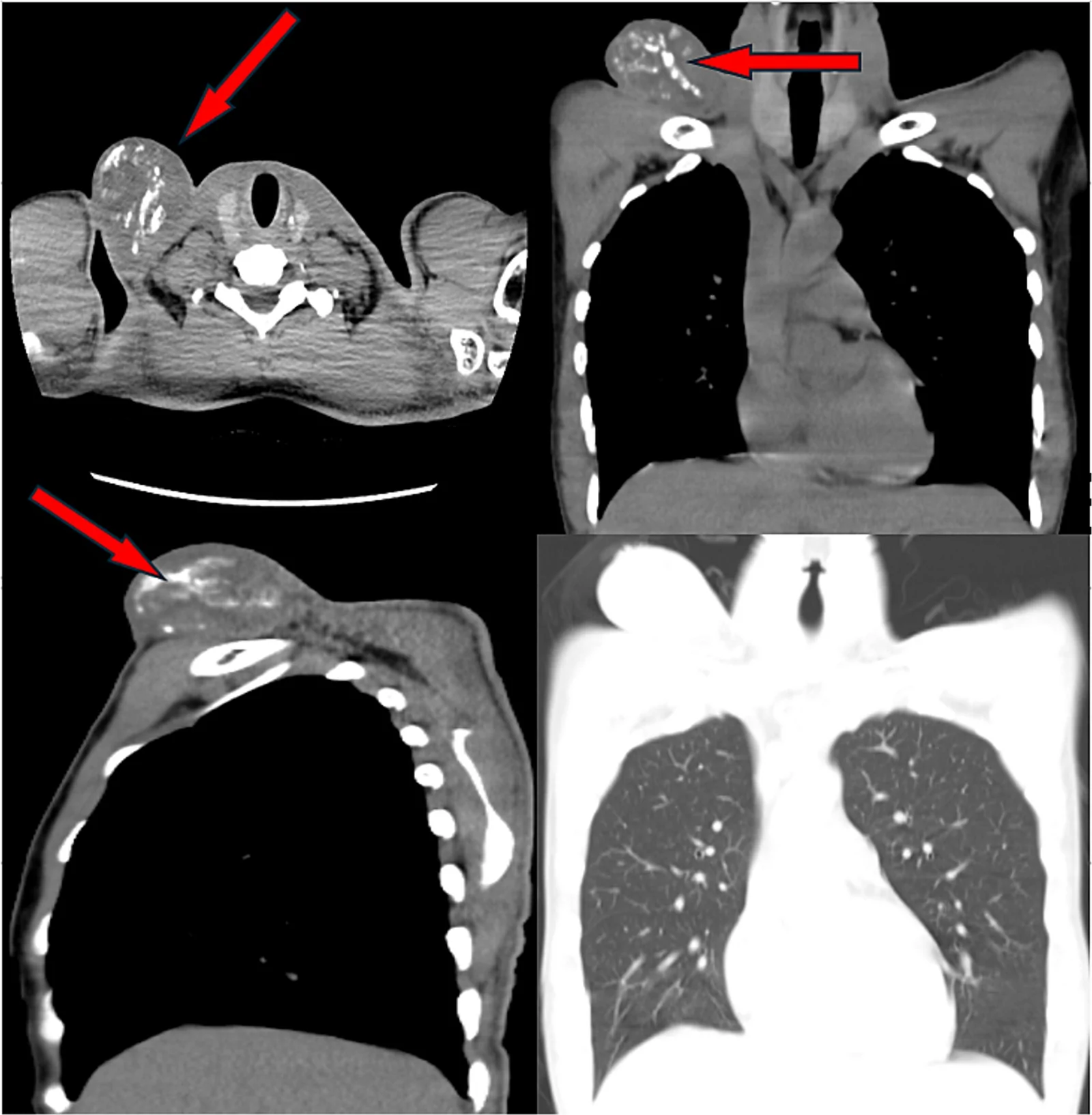
Chest CT images in axial, coronal, and sagittal planes demonstrating a well-defined encapsulated subcutaneous mass with internal calcifications in the right upper anterior chest wall (arrows). The lesion is separate from adjacent thoracic structures and shows no intrathoracic extension. Lung-window images demonstrate normal bilateral pulmonary parenchyma.

Based on clinical and radiological findings, the differential diagnosis included both benign and malignant soft-tissue neoplasms. Given the diagnostic uncertainty, complete excisional biopsy was performed for both diagnostic and therapeutic purposes (Fig. 4). The postoperative course was uneventful.

**Figure 4:**
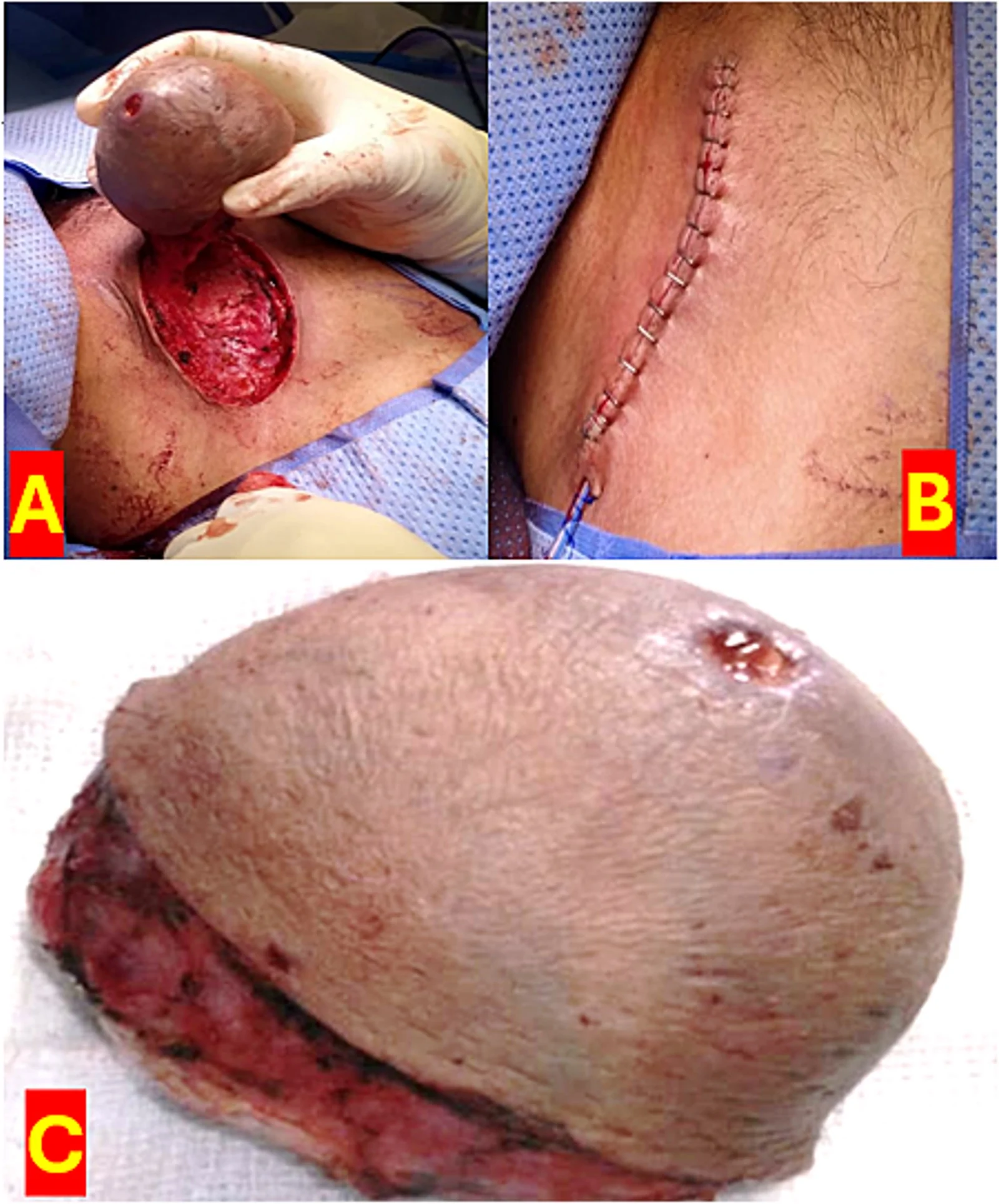
Intraoperative and gross specimen photographs. (A) Complete excision of the right subclavicular chest wall mass through an elliptical skin incision with adequate surgical margins; the lesion remains attached to its vascular pedicle prior to removal. (B) Operative field following tumor resection demonstrating primary wound closure with a surgical drain *in situ*. (C) Gross appearance of the excised specimen, showing a well-circumscribed subcutaneous mass with overlying skin ulceration.

Histopathological examination confirmed chronic fibrocaseous granulomatous inflammation consistent with tuberculous lymphadenitis, establishing the diagnosis of a giant tuberculous lymph node.

Following the diagnosis, the patient was treated with standard anti-tuberculous therapy for six months, with satisfactory clinical recovery and no evidence of recurrence during follow-up.

Case 2: A 35-year-old male presented with a 2–3-month history of a gradually enlarging mass over the right posterolateral chest wall. The swelling was initially painless but became progressively painful with enlargement, particularly during movement of the right shoulder. The patient reported intermittent fever and sweating but denied cough, dyspnea, hemoptysis, weight loss, or previous TB exposure. He had no significant past medical or surgical history.

Physical examination revealed a large right posterolateral chest wall mass measuring ~15 × 20 cm, extending from just below the inferior angle of the scapula to the ipsilateral costal margin (Fig. 5). The lesion was tense, fluctuant, and fixed to the underlying tissues, with intact overlying skin and no local inflammatory changes. Reduced air entry was noted over the right basal lung zone. No peripheral lymphadenopathy was present.

**Figure 5:**
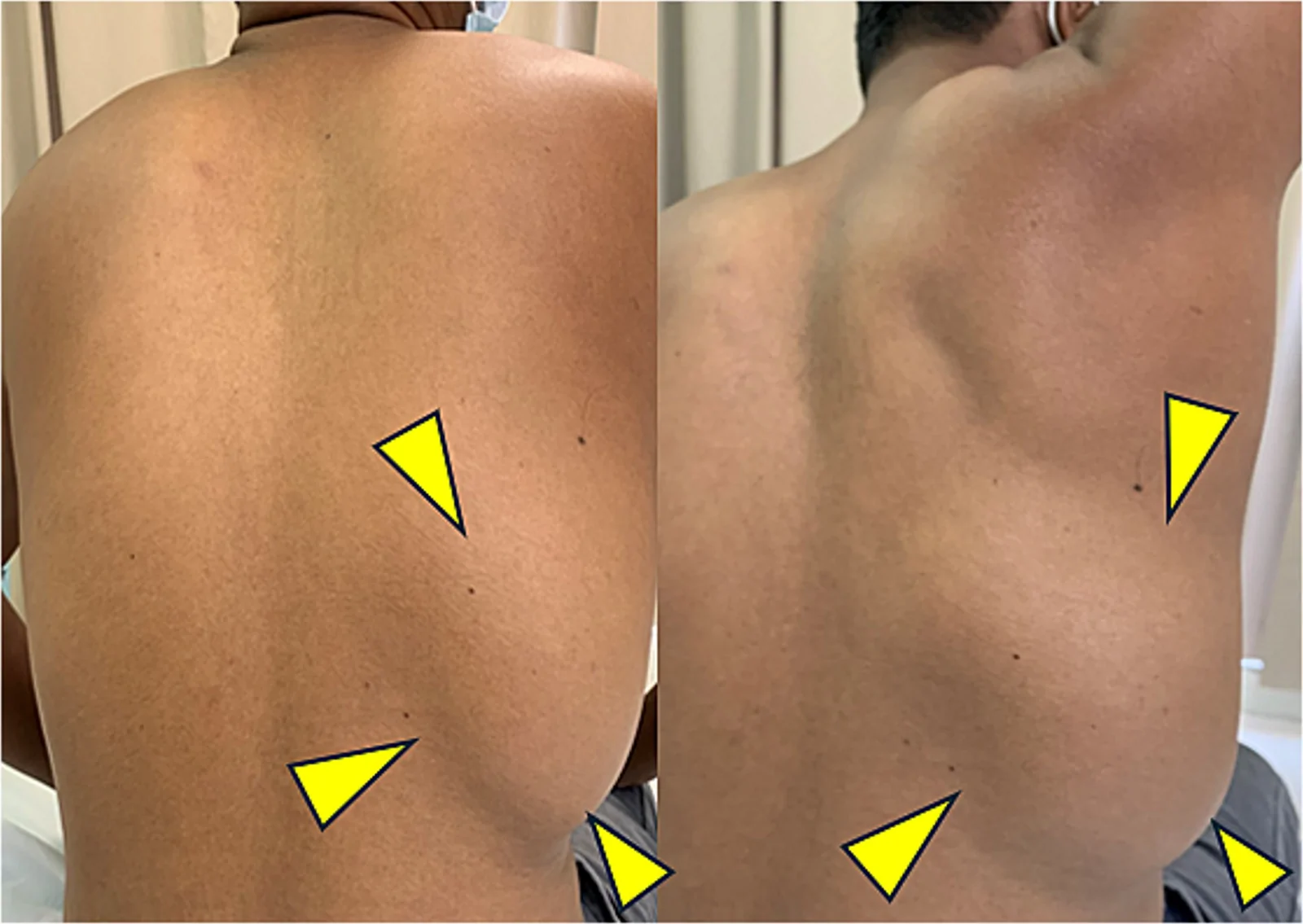
Clinical photographs demonstrating a large right posterolateral chest wall swelling extending from below the inferior angle of the scapula to the ipsilateral costal margin (arrowheads). The overlying skin appears intact.

Routine laboratory investigations were largely unremarkable apart from mild leukocytosis (12.8 × 10^9^/l), thrombocytosis (552 × 10^9^/l), elevated erythrocyte sedimentation rate (33 mm/h), and elevated C-reactive protein (10.8 mg/l). Sputum acid-fast bacilli smears were negative on three consecutive samples.

Chest radiography demonstrated a soft tissue bulge along the right lateral chest wall without underlying pulmonary or pleural abnormalities (Fig. 6). The remainder of the radiographic examination was unremarkable.

**Figure 6:**
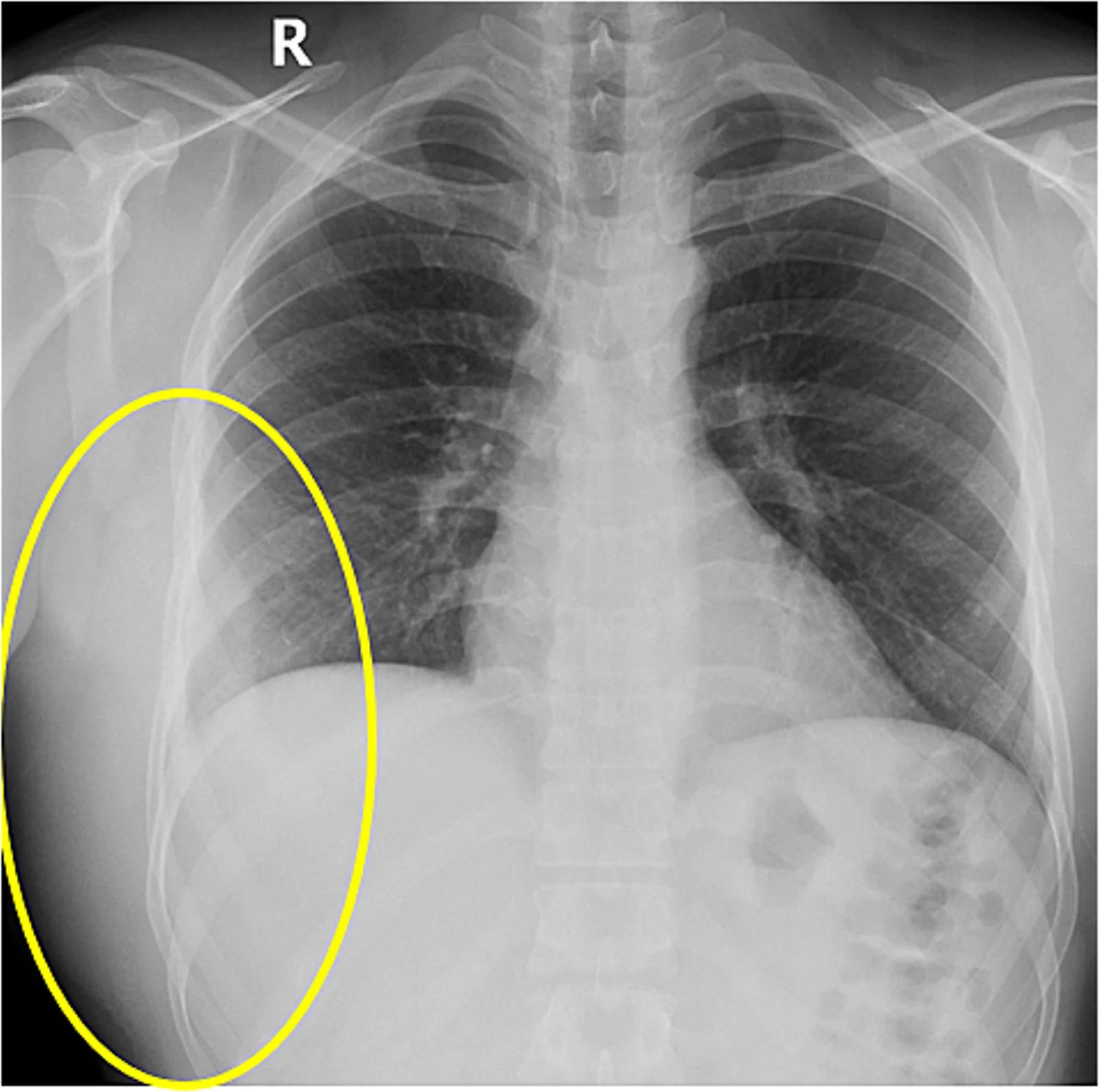
Posteroanterior chest radiograph demonstrating a prominent soft tissue bulge along the right lateral chest wall (encircled). No associated pulmonary parenchymal or pleural abnormalities are identified.

Contrast-enhanced CT of the chest revealed a large rim-enhancing cystic collection measuring 11 × 8.6 × 5.6 cm within the right posterolateral chest wall deep to the back muscles, with extension into the overlying soft tissues. Adjacent pleural thickening was also noted. No focal pulmonary lesions were identified, although mild mediastinal lymphadenopathy was present (Fig. 7).

**Figure 7:**
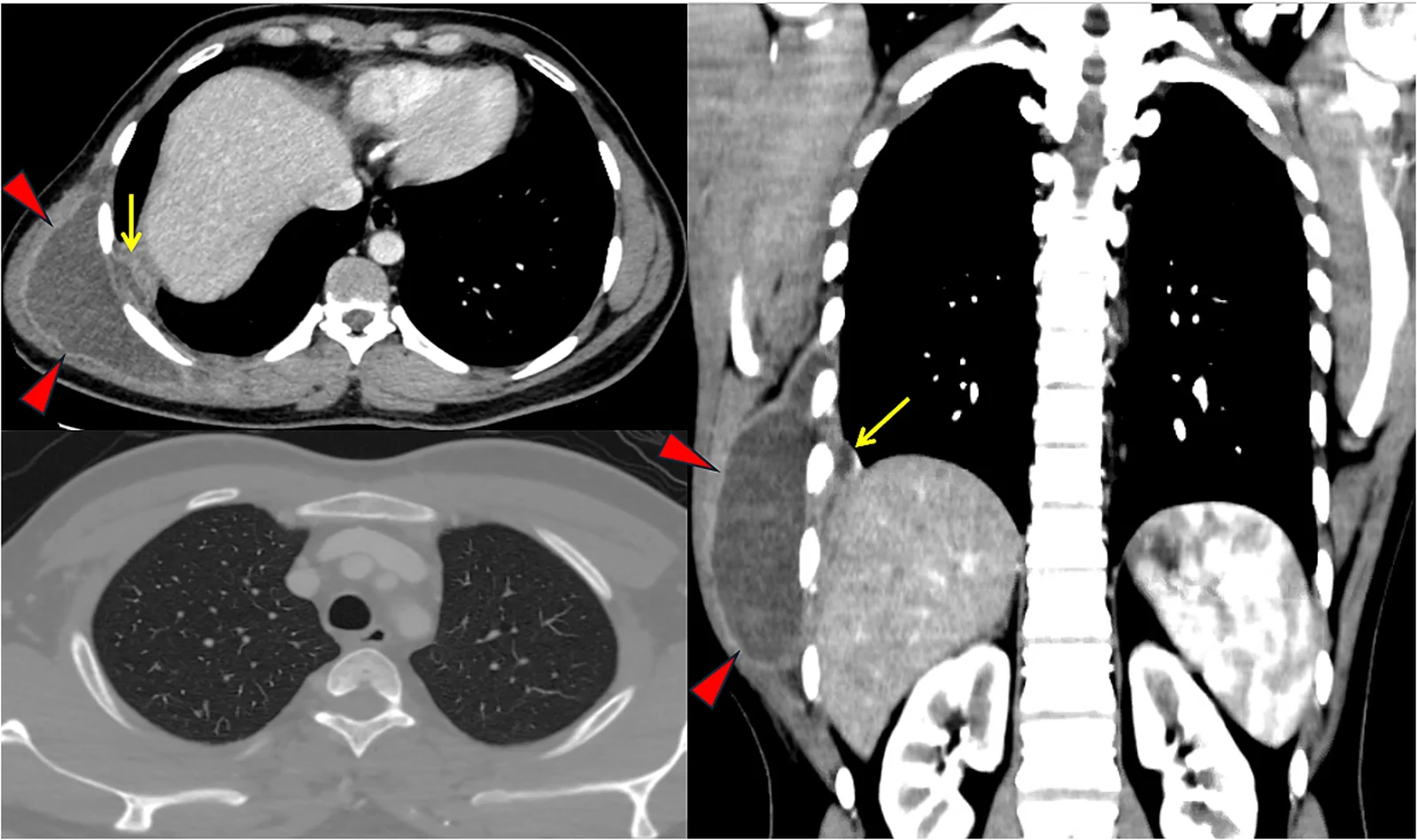
Contrast-enhanced CT images demonstrating a large rim-enhancing cystic collection within the right posterolateral chest wall deep to the back muscles, with extension into the overlying soft tissues (arrowheads). Adjacent pleural thickening is present (arrow). Both lungs appear normal without focal parenchymal lesions.

Given the investigation findings consistent with a large chest wall collection, the patient underwent incision and drainage of the abscess with evacuation of copious purulent material. Debridement of the cavity was performed, and pus, and soft-tissue specimens were obtained for microbiological, and histopathological analysis. A closed-suction drain was placed before wound closure (Fig. 8).

**Figure 8:**
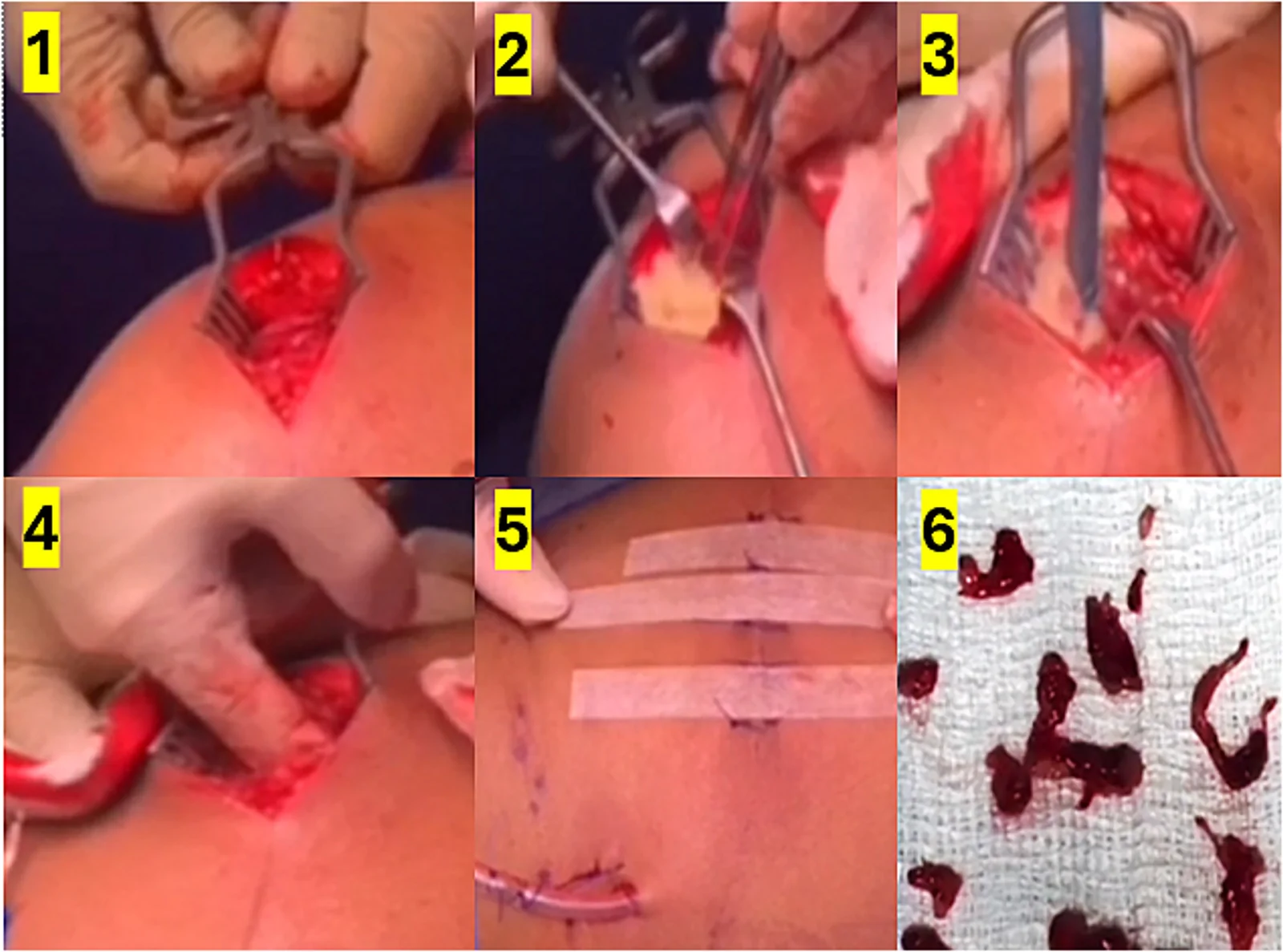
Operative photographs demonstrating surgical management of the chest wall abscess. (1) Drainage incision. (2) Evacuation of thick, opaque pus following entry into the abscess cavity. (3) Suction-assisted drainage of the collection. (4) Blunt finger dissection of the cavity to disrupt internal septations and obtain tissue samples for histopathological examination. (5) Wound closure with a closed-suction drain *in situ*. (6) Soft-tissue specimens obtained for pathological analysis.

Polymerase chain reaction (PCR) testing of the drained pus was positive for *M. tuberculosis*, while histopathological examination revealed necrotizing granulomatous inflammation consistent with TB (cold abscess), confirming the diagnosis of chest wall TB. A small, localized recollection developed 1 month after the initial drainage and was successfully managed by repeat drainage. The patient subsequently completed a 6-month course of anti-tuberculous therapy and achieved complete recovery without recurrence.

## Discussion

### Clinical presentation

Primary TB of the chest wall is rare and diagnosis in most cases is challenging because the lesions grossly simulate pyogenic abscess or tumor. Tuberculous abscesses of the chest wall can involve the sternum, costochondral junctions, ribs shafts, costovertebral joints, and the vertebrae. In a case series of 15 cases, the lesions were fistulized to the skin in 40% [6]. They usually occur as a solitary lesion, most frequently at the margins of the sternum and in the shafts of the ribs [4, 5]. In a series of 32 cases of chest wall TB, only 59.4% of patients had a current or previous history of TB [3].

The present report highlights two uncommon manifestations of chest wall TB. In the first case, TB presented as a solitary chest wall mass with central ulceration highly suggestive of a soft tissue tumor. In the second case, the disease manifested as a large, isolated chest wall abscess on the lateral chest wall without bone involvement, and without evidence of active or previous pulmonary TB.

These presentations underscore the ability of chest wall TB to mimic both neoplastic and infectious conditions, often delaying diagnosis.

### Diagnosis and differential diagnosis

Radiologic imaging is important in the assessment of chest wall TB, particularly for determining anatomic origin and extent, response to therapy, and recurrence. CT imaging plays an important role in the evaluation of this chest wall abnormality due to its excellent spatial resolution, including depiction of both osseous and soft-tissue structures, and providing valuable information on the functional status of the lungs [3, 4]. The absence of constitutional symptoms and normal pulmonary imaging may further complicate clinical suspicion. Consequently, histopathological examination and microbiological confirmation remain essential diagnostic tools [1, 3, 6].

Microbiological examination of the serosanguineous discharge, including acid-fast bacillus staining, mycobacterial culture, or PCR testing for *M. tuberculosis*, was not performed preoperatively. Similarly, an interferon-gamma release assay (IGRA) was not obtained. At presentation, the lesion’s clinical and radiological characteristics, particularly its size and appearance, were considered more suggestive of a soft-tissue neoplasm, and surgical excision was therefore planned for definitive diagnosis and treatment.

The elevated CRP level (36.7 mg/l) and the presence of serosanguineous discharge could have raised the possibility of an infectious or inflammatory process. However, CRP elevation is nonspecific and may also occur in neoplastic lesions, particularly in association with tissue inflammation or necrosis. There were insufficient preoperative clinical features to strongly favor TB or another infectious etiology over neoplasia. In retrospect, microbiological examination of the discharge should be considered in similar cases, as it may provide an additional diagnostic clue when an infectious etiology is present. Likewise, although IGRA cannot distinguish latent from active TB and cannot establish the diagnosis of chest wall TB, a positive result may increase clinical suspicion and prompt further microbiological investigation as well as appropriate infection-control planning.

These cases also highlight the infection-control implications of delayed recognition of extrapulmonary TB. In Case 1, standard infection-control precautions were applied because a neoplastic process was considered the most likely diagnosis and there was no evidence of pulmonary involvement. In Case 2, where a cold abscess was included in the differential diagnosis, the procedure was performed in a negative-pressure operating room with enhanced precautions according to institutional policy. Following confirmation of TB in both cases, the Infection Control Department was notified, and the patients were managed according to institutional TB protocols. Owing to the extrapulmonary nature of the disease and the absence of pulmonary involvement, no occupational exposure requiring additional healthcare-worker screening or follow-up evaluation was identified.

Although tissue diagnosis remained necessary for definitive confirmation, greater preoperative suspicion of TB may facilitate earlier microbiological investigation and appropriate infection-control measures.

### Management

Treatment of chest wall TB is controversial and there is no consensus on the optimal treatment. Few studies suggest that medical treatment by antituberculosis chemotherapy alone is effective, while most others believe that combination of complete excisional resection for a small, localized lesion, or aggressive drainage and debridement for a large abscess, and medical therapy is required to prevent recurrence [3, 4, 7].

Although World Health Organization (WHO) recommends a standard 6-month regimen, according to clinical presentation, bacillary load, and response to anti-tuberculous medical therapy, the treatment can be extended up to 9–12 months [4].

In the first case, excisional resection of the localized solitary lesion was an essential diagnostic and therapeutic procedure, while in the second case, the extended drainage and debridement of the giant abscess was the cornerstone of treatment. Both cases underwent appropriate surgical interventions and timely referral to the infection control team to receive the appropriate medical treatment. There were no recurrences during the 5-year follow-up period.

Chest wall TB is an uncommon entity that may present as a tumor-like chest wall mass or as an isolated cold abscess in the absence of pulmonary disease. These atypical manifestations should be considered in the differential diagnosis of chest wall lesions, particularly in low-incidence TB settings where clinical suspicion may be low. Histopathological and microbiological confirmation remain essential for diagnosis, and a combination of appropriate surgical intervention and anti-tuberculous therapy can result in excellent clinical outcomes.

## Data Availability

All data supporting the findings of this report are included within the article.
